# The Role of microRNAs in the Biology of Rare Diseases

**DOI:** 10.3390/ijms12106733

**Published:** 2011-10-11

**Authors:** Marco Salvatore, Armando Magrelli, Domenica Taruscio

**Affiliations:** National Centre for Rare Diseases, Istituto Superiore di Sanità Viale Regina Elena, Rome 299-00161, Italy; E-Mails: marco.salvatore@iss.it (M.S.); armando.magrelli@iss.it (A.M.)

**Keywords:** microRNA, rare disease, biomarker, hepatoblastoma, multiple osteochondromas, Sezary syndrome, Hailey-Hailey disease, Rett syndrome

## Abstract

Rare diseases (RD) are characterized by low prevalence and affect not more than five individuals per 10,000 in the European population; they are a large and heterogeneous group of disorders including more than 7,000 conditions and often involve all organs and tissues, with several clinical subtypes within the same disease. Very often information concerning either diagnosis and/or prognosis on many RD is insufficient. microRNAs are a class of small non-coding RNAs that regulate gene expression at the posttranscriptional level by either degrading or blocking translation of messenger RNA targets. Recently, microRNA expression patterns of body fluids underscored their potential as noninvasive biomarkers for various diseases. The role of microRNAs as potential biomarkers has become particularly attractive. The identification of disease-related microRNAs is essential for understanding the pathogenesis of diseases at the molecular level, and is critical for designing specific molecular tools for diagnosis, treatment and prevention. Computational analysis of microRNA-disease associations is an important complementary means for prioritizing microRNAs for further experimental examination. In this article, we explored the added value of miRs as biomarkers in a selected panel of RD hitting different tissues/systems at different life stages, but sharing the need of better biomarkers for diagnostic and prognostic purposes.

## 1. Introduction

According to the Regulation EC n.141/2000, rare diseases (RD) are defined on the basis of low prevalence and affect not more than five individuals per 10,000 in the European population [1].

In the USA the Orphan Drug Act of 1983 defines a RD as “any disease or condition that affects less than 200,000 people in the United States”. In Japan, the Japanese Medicines Act of 1993 defines a rare disease as a condition affecting no more than 50,000 people in the country.

They are a large and diverse group of disorders, estimated to include more than 6000 conditions, often involving all organs and tissues with several clinical subtypes within the same disease. Several groups of RD include congenital malformations, metabolic defects, rare tumors, skin diseases, syndromes involving body systems such as the nervous, immune, excretory, reproductive, cutaneous ones. About 80% of RD have genetic origins, involving one or several genes or chromosomal abnormalities; in many cases the clinical phenotype and prognosis appear to be modulated by yet unknown factors, such as different mutations in the same gene(s) or also epigenetic factors.

The difficulty in obtaining the correct diagnosis is the first dramatic hurdle for rare disease patients. It has been estimated that about 30% of RD are still lacking a diagnostic definition; moreover, many RD cases get an accurate diagnosis late, even after five years or more from disease recognition. Late diagnoses delay specific treatments and might have severe, irreversible, debilitating and life-threatening consequences [2].

Accurate and timely diagnosis is often of principal importance for prevention and properly targeted treatment; where these are unavailable, timely diagnosis is always important for protecting the patients’ quality of life. However, due to problems associated with rarity (low number of patients, weak interest by research institutions, *etc*.), information on many RD are insufficient concerning either diagnosis and/or prognosis.

Biomarkers are measurable bio-parameters that can be used to monitor disease progression, prognosis and drug response, thus optimizing the choice of appropriate and often personalized therapies. Their identification in RD is often impaired by the low number of cases available for each disease, making a sound evaluation of sensitivity, specificity, and predictive value difficult. In particular, some groups of RD, such as inherited disorders and rare tumors, are insufficiently elucidated from the standpoint of pathogenesis and biomarkers.

Furthermore, it is now well ascertained that RD suffer from the relative lack of information from natural history studies of diseases. The specificities of rare diseases—limited number of patients and scarcity of relevant knowledge and expertise—can be tackled through a global approach. Very recently**,** an international effort (International Rare Disease Research Consortium, IRDiRC) has been formed under the auspices of the US National Institutes of Health (NIH) and the European Commission (EC) with the ambitious goal of developing a diagnostic tool for every known rare disease by 2020, along with new therapies to treat 200 of them [3].

## 2. microRNA: Function and Role

microRNAs (miRs) are regulated and transcribed like protein coding genes. Subsequent miR biogenesis involves discrete processing and transport steps, whereby the active moiety of 20–22 nucleotides is excised from a longer RNA precursor that exhibits specific hairpin structure. Finally, these 20–22 nucleotides are incorporated into a composite machinery, which promotes partial duplex formation between the short RNA and the untranslated regions (UTR) of targeted mRNAs, resulting typically in translational silencing in mammals.

Although the total number of miR remains controversial and their roles are only beginning to be defined, miR expression analyses indicate that diverse tumors display miRs expression profiles (for mature and/or precursor miRs) significantly different from normal tissue. Furthermore, miRs are emerging as highly tissue-specific biomarkers with potential clinical applicability for defining the cancer origin of metastases. They have been appreciated in various fundamental biological processes such as cell proliferation [4], stem cell division [5] and apoptosis [6]. Recently, it has been revealed that altered expression of specific miR genes contributes to the initiation and progression of cancer [7–9]. Translational studies suggest that their signature may be useful in categorizing and predicting the course of an increasing number of human pathologies.

## 3. Diagnostic Utility of miRs and Efficacy as Potential Biomarkers

Future research targets integrate sets of new biomarkers to be developed with a system biology approach and in combination with bioinformatics tools, to devise powerful diagnostic and prognostic algorithms. Ideally, biomarkers should be easily accessible such that they can be sampled non-invasively. Therefore biomarkers that can be sampled from body fluids, such as serum or urine, are particularly desirable. In recent years it has become clear that miRs could represent new effective biomarkers.

miRs as a diagnostic tool might be used to detect an increased risk of acquiring RD and cancer by studying disease associated variations of miR sequences (like miR-146a in thyroid cancer) [10]; to diagnose early-stage cancer by determining miR expression profiles in the blood, like miR-141 in prostate cancer [11]; to distinguish tumor from normal tissue in fine-biopsy specimens by miR expression profiling [7]; and to predict clinical outcome, like in leukemia [12]. Furthermore, human genetic variation potentially influences many medical conditions. Genetic variation can either broadly affect miR biogenesis or specifically hit a miR or its target. The resulting misregulation of miRs and their targets is potentially crucial to pathogenesis [13,14]. In genome wide association studies (GWAS), copy number variation (CNV) and single nucleotide polymorphisms (SNPs) within the sequences of human miRs and their targets have been shown to have impact on various phenotypes including blood pressure, drug resistance, mental disorder, gastric mucosal atrophy and Parkinson disease [15].

Usefulness of miRs as new effective biomarkers is due to the fact that (i) miRs expression is known to be aberrant in cancer; (ii) miRs expression profiles are pathognomonic, or tissue-specific; (iii) over expression or lack of expression of specific miRs appear to correlate with clinically aggressive or metastatic phenotypes; (iv) miRs are remarkably stable molecules being well preserved in formalin fixed, paraffin embedded tissues, fresh snap frozen specimens and body fluids.

Very few studies have been reported on possible alternative mechanisms underlying specific RDs onset and clinical manifestations and progression. This causes the lack of diagnostic/prognostic markers that are strongly needed for an adequate follow-up of patients, currently based only on classical screenings.

The identification of miRs associated with human diseases is an important goal of biomedical research. Recently, a number of computational methods have been developed to predict or prioritize diseases-related genes. Most approaches are based on the idea that dysfunctions of functionally related genes tend to be associated with phenotypically similar diseases. These genes linked to similar diseases usually interact with each other or participate in the common biological modules. Network-based approaches have also been employed to predict or prioritize new candidate disease genes based upon network linkages with known disease genes. These approaches typically start with constructing a gene-gene association network based on one or more types of genomic and proteomic information, and then prioritize candidate protein-coding genes based on network proximity to known disease-related genes [16].

## 4. microRNA and Rare Diseases

Even though few studies are available on miRs and their association with RDs, some evidence is emerging on their role in controlling pathways involved in RDs onset and/or progression. In this paragraph we briefly review the latest findings on selected RDs hitting different tissues/systems at different life stages, but sharing the need of better biomarkers for diagnostic and prognostic purposes (Table 1).

*Duchenne muscular dystrophy* (DMD, OMIM 310200) is a lethal X-linked disorder caused by mutations in the dystrophin gene, which encodes a cytoskeletal protein, dystrophin. It has been recently demonstrated that the serum levels of muscle-specific miRs (miR-1, miR-133, miR-206) are released in to the bloodstream of DMD patients [17]; increased level of miRs has also been observed in the dystrophin-deficient mouse model, as well as the canine X-linked muscular dystrophy in Japan dog model [18]. Recent works have shown that among genes which are important for proper muscle development and function, miRs play a crucial role [19–21]. Moreover, altered levels of miRs were found in several muscular disorders such as myocardial infarction [22], DMD, and other myopathies [23,24]. Yuasa and colleagues [25] showed that miR-206 expression was increased after cardiotoxin-induced muscle regeneration and that miR-206 contributes to muscle regeneration. Interestingly, it has been showed that the expression levels of miR-206 in DMD patients are not increased [23] or that the increase is not as large as in mouse model [24].

*Amyotrophic lateral sclerosis* (ALS, OMIM 105400) is a neurodegenerative disease characterized by loss of motor neurons, denervation of target muscles, muscle atrophy, and paralysis. Williams and colleagues showed that a key regulator of this RD is the miR-206, a skeletal muscle-specific miR that is dramatically induced in a mouse model of ALS. Mice that are genetically deficient in miR-206 form normal neuromuscular synapses during development, but deficiency of miR-206 in the ALS mouse model accelerates disease progression. MiR-206 is required for efficient regeneration of neuromuscular synapses after acute nerve injury, which probably accounts for its salutary effects in ALS [26].

*Sezary syndrome* (SS) is a rare and aggressive leukemic variant of cutaneous T-cell lymphoma (CTCL) characterized by the presence of neoplastic lymphocytes named Sezary cells, in the skin, lymph nodes and peripheral blood [27]. Expression patterns of miRs and their role in the pathogenesis of SS have only recently been addressed. Ballabio and colleagues reported that 104 out of 114 SS-associated miRs were down-regulated and that their expression pattern was consistent with previously reported genomic copy number abnormalities Altered level of miR-223 was able to discriminate SS samples from healthy controls; down-regulation of intronically encoded miR-342 plays a role in the pathogenesis of SS by inhibiting apoptosis, and describe a novel mechanism of regulation for this miR [28]. Another study conducted on a cohort of 22 SS patients, the expression profile of 470 miRs, conducted to the identification of 45 miRs differentially expressed between SS and controls. Using predictive analysis, a list of 19 miRs, including miR-21, miR-214, miR-486, miR-18a, miR-342, miR-31 and let-7 members were also found. Moreover, a signature of 14 miRs including again miR-21, potentially able to discriminate patients with unfavorable and favorable outcome, was identified [29]. van der Fits and colleagues showed that miR-21 expression is increased in SS cells when compared with CD4+ T cells from healthy donors. Silencing of miR-21 in SS cells results in increased apoptosis, suggesting a functional role for miR-21 in the leukomogenic process, thus representing a putative therapeutic target for the treatment of SS [30].

*Rett syndrome* (RTT, Online Mendelian Inheritance in Man Database 312750) is a complex neurological disorder that has been associated with mutations in the gene coding for the methyl CpG binding protein 2 (Mecp2). This X-linked neurological disorder causes a severe phenotype in one out of every 10,000–15,000 live births, making it the second most frequent cause of mental retardation in females. Very recently it has been examined the possible effects of miR misregulation caused by Mecp2 absence in a mouse model of RTT. Using miR expression microarrays, it was observed that the brain of Rett syndrome mice undergoes a disruption of the expression profiles of miRs. Among the significantly altered miRs (26%, 65 of 245), overall downregulation of these transcripts was the most common feature (71%), while the remaining 30% were upregulated. In particular it was demonstrated that the most commonly disrupted miRs were miR-146a, miR-146b, miR-130, miR-122a, miR-342 and miR-409 (downregulated) and miR-29b, miR329, miR-199b, miR-382, miR-296, miR-221 and miR-92 (upregulated) [31].

*Multiple osteochondromas* (MO, OMIM 133700) are characterized by a large spectrum of germline mutations scattered along EXT1/EXT2 genes, by the presence of a significant percentage of patients without alterations in the EXT genes and a large phenotypic intra- and interfamilial variability. The molecular basis of MOs genetic and clinical heterogeneity are currently unknown. This leads to the lack of appropriate diagnostic/prognostic markers as well as of therapeutic options for both benign and malignant lesions. 45 miRs have been identified as differentially expressed in MO patients, and classified them into 4 classes according to their expression compared to articular and/or growth plate cartilage. In particular a signature of 8 miRs (namely: miR-21, miR-140, miR-145, miR-214, miR-195, miR-199a, miR-451, and miR-483) was able to distinguish healthy growth control from MO patients. Compared to microarray data clustering, this finding allows to more accurately identify miRs specifically related to pathologic condition of growth plate cartilage. Results indicate that miRs differentially expressed may hamper the molecular signaling responsible for normal proliferation and differentiation of chondrocytes, thus contributing to pathogenesis and clinical outcome [32].

*Hailey-Hailey* disease (HHD, OMIM 169600) is an autosomal dominant disorder characterized by suprabasal cutaneous cell separation leading to the development of erosive and oozing skin lesions. While a strong relationship exists between mutations in the gene ATP2C1 and HHD, a poor understanding of how these mutations affect manifestations of the disease is available. A specific signature discriminating lesion from normal skin was recently found. The authors found, that miR-181a, miR-125b, and miR-99a expression increases in HHD-lesion derived keratinocytes. Interestingly, the expression of miR-181a, miR-125b and miR-99a has an anti-proliferative effect in several cell types [33–35]. Additionally, miR-106a aberrantly expressed in primary tumors and cancer derived cell lines [36] was found to be downregulated in HHD-lesion derived keratinocytes. Collectively, these results suggest that a perturbed expression for these miRs in HHD skin lesion may contribute to hypo-proliferation of keratinocytes. Computational analysis to identify lesion-specific miRs with the most interesting and promising pathogenetic function identified miR-125b as the most significantly induced [37,38]. MiR-125b has been found to be also involved in the TNF-alpha pathway and deregulated in psoriasis and atopic eczema [39].

The *Hepatoblastoma* (HB, OMIM 114550) is a rare pediatric liver tumor with an incidence in Western countries of 1.5 cases per million of individuals younger than 15 years. Despite this, it is the most common hepatic malignancy during childhood. Although specific chromosome aberrations have not been linked with prognosis or casual factors, genetic disorders have been described in chromosomes 1q, 2q, 7q, 8, 17q and 20 as well as activation of Wnt signaling pathway through mutations in its central mediator beta-catenin. Whole genome analysis of miRs showed that 4 miRs were significantly upregulated in the tumor compared with the nonmalignant tissue (miR-214, miR-199a, miR-150, and miR-125a) and one was significantly downregulated (miR-148a). Among these, miR-214 has been shown to be deeply involved in controlling the level of PTEN protein, a negative regulator of the PI3K signaling whose alteration has been shown to give rise to different cancers [40]. Finally, recent work showed a functional relationship between Keratin 19 and miR-492 that may play an important role in the progression of malignant embryonal liver tumors. The author stated that co-regulation of miR-492 and keratin 19 expression in HB, occurs predominantly in metastatic tumors, providing novel experimental evidence that miR-492 could represent a marker of aggressive tumor behavior. MiR-492 and its associated targets might serve as promising biomarker candidates in both diagnostic and therapeutic strategies aiming at improving outcome of HB [41].

## 4. Perspectives

The use of miRs as biomarkers is a promising field. Their basic biology is still being elucidated and questions regarding tissue-specific functions and targets, developmental time of expression, regulation and evolutionary role are being explored. With their key roles in gene expression regulation, particularly during cell differentiation and tissue genesis, miRs are probably critical in cancer initiation and progression, but more research studies are needed to explore their impact as clinical biomarkers for cancer and disease diagnosis, prognosis and prediction of therapeutic response. Outputs of this new field will be the identification of molecular markers to detect potential therapeutic targets, new biomarkers which could improve diagnosis and management, optimize patients’ follow-up and, in turn, improve their quality of life. The development of minimally invasive tests for the detection and monitoring of RD can reduce their worldwide health burden. Although conventional strategies for blood-based biomarker discovery (e.g., using proteomic technologies) have shown promise, the development of clinically validated cancer detection markers remains an unmet challenge for many common human cancers. It is strongly believed that studies on the biological basis of the above mentioned pathologies will improve their understanding and support the development of perspective therapies. The identification of others disease-related miRs is extremely important for understanding the pathogenesis of diseases at the molecular level and is critical for designing specific molecular tools for diagnosis, treatment and prevention.

## 5. Conclusions

More than 1500 transcribed miRs have been identified in the human genome since their discovery. miR analysis is a promising tool for identifying new biomarkers, as well as for predicting response to cancer and rare diseases therapy. Recently, several reports suggest that cell-free circulating miRs are detectable in serum/plasma and the levels of tumor-derived miRs are altered in patients with tongue cancer, lung cancer, prostate cancer, ovarian cancer, and colorectal cancer (reviewed in this issue). These findings suggest that blood-based miRs could emerge as revolutionary sources of biomarker for diagnosis. Further understanding of their functional roles will open up new opportunities in developing revolutionary therapeutic strategies. Although we are just beginning to understand the specific contribution of miRs in RD, a larger sample sets including long-term clinical data are urgently required for future studies. Finally, major efforts need to be applied on standardization of isolation, quantification and normalization strategies before any of the novel miR biomarkers is applicable for clinical practice.

## Figures and Tables

**Table 1:** Rare disease and microRNAs.

Rare Disease	miR	Note	References
Duchenne muscular dystrophy	miR-206, miR-181, miR-1, miR-133, miR-29	Up- and downregulated	[17–25]
Amyotrophic lateral sclerosis	miR-206	Downregulated	[26]
Sézary syndrome	miR-21, miR-214, miR-486, miR-18a, miR-342, miR-31, let-7, miR-233, miR-199a	Up- and downregulated	[27–30]
Rett syndrome	miR-146a, miR-146b, miR-130, miR-122a, miR-342, miR-409 miR-29b, miR-329, miR-199b, miR-382, miR-296, miR-221, miR-92	Up- and downregulated	[31]
Multiple osteochondromas	miR-21, miR-140, miR-145, miR-214, miR-195, miR-451, miR-483	Up- and downregulated	[32]
Hailey-Hailey disease	miR-181a, miR-125b, miR-99, miR-106a	Up- and downregulated	[33–39]
Hepatoblastoma	miR-214, miR-199a, miR-150, miR-125a, miR-148a, miR-492	Up- and downregulated	[40,41]
